# Spatial distribution and determinants of intimate partner violence among reproductive-age women in Ethiopia: Spatial and Multilevel analysis

**DOI:** 10.1186/s12905-021-01218-3

**Published:** 2021-02-25

**Authors:** Dessie Abebaw Angaw, Alemakef Wagnew Melesse, Bisrat Misganaw Geremew, Getayeneh Antehunegn Tesema

**Affiliations:** Department of Epidemiology and Biostatistics, Institute of Public Health, College of Medicine and Health Sciences, University of Gondar, Gondar, Ethiopia

**Keywords:** Intimate partner violence, Ethiopia, Multilevel analysis, Spatial analysis

## Abstract

**Background:**

Intimate partner violence is a serious global public health problem particularly in low-and middle-income countries such as Ethiopia where women's empowerment is limited. Despite the high prevalence of intimate partner violence in Ethiopia, there is limited evidence on the spatial distribution and determinants of intimate partner violence among reproductive-age women. Exploring the spatial distribution of intimate partner violence is crucial to identify hotspot areas of intimate partner violence to design targeted health care interventions. Therefore, this study aimed to investigate the spatial distribution and determinants of intimate partner violence among reproductive-age women in Ethiopia.

**Methods:**

A secondary data analysis was done based on the 2016 Ethiopian Demographic and Health Survey (EDHS) data. A total weighted sample of 6090 reproductive-age women were included in the study. The spatial scan statistical analysis was done to identify the significant hotspot areas of intimate partner violence. A multilevel binary logistic regression analysis was fitted to identify significant determinants of intimate partner violence. Deviance, Intra-cluster Correlation Coefficient (ICC), Median Odds Ratio, and Proportional Change in Variance (PCV) were used for model comparison as well as for checking model fitness. Variables with a p-value less than 0.2 were considered in the multivariable analysis. In the multivariable multilevel analysis, the Adjusted Odds Ratio (AOR) with 95% Confidence Interval (CI) were reported to declare statistical significance and strength of association between intimate partner violence and independent variables.

**Results:**

The spatial analysis revealed that the spatial distribution of intimate partner violence was significantly varied across the country (Moran’s I = 0.1007, p-value < 0.0001). The SaTScan analysis identified a total of 192 significant clusters, of these 181 were primary clusters located in the Benishangul-Gumuz, Gambella, northwest Amhara, and west Oromia regions. In the multivariable multilevel analysis; women aged 45–49 years (AOR = 2.79, 95% CI 1.52–5.10), women attained secondary education (AOR = 0.61, 95% CI 0.38–0.98), women in the richest household (AOR = 0.58, 95% CI 0.35–0.97), > 10 family size (AOR = 3.85, 95% CI 1.41–10.54), and high community women empowerment (AOR = 0.66, 95% CI 0.49–0.8)) were significantly associated with intimate partner violence.

**Conclusions:**

Intimate partner violence among reproductive-age women had significant spatial variation across the country. Women's age, education status, family size, community women empowerment, and wealth status were found significant determinants of intimate partner violence. Therefore, public health programs should design targeted interventions in identified hot spot areas to reduce the incidence of intimate partner violence. Besides, health programmers should scale up public health programs designed to enhance women's autonomy to reduce the incidence of intimate partner violence and its consequences.

## Background

According to the World Health Organization (WHO), Intimate Partner Violence (IPV) is defined as any behavior within an intimate partner that causes physical, psychological, or sexual harm [1]. IPV is the commonest form of violence that encompasses physical, sexual, and emotional violence [2–4]. Globally, an estimated 11 million women experience sexual, physical, or psychological violence by their intimate partner in their lifetime [5]. Overall, 30% of women experienced physical or sexual harassment throughout their lifetime by an intimate partner ranged from 24.6% in the West Pacific to 36.6% in Africa. However, in low-and middle-income countries, it reached up to 70% [2, 6, 7]. In Ethiopia, 59% and 42% of women faced sexual and physical violence by their intimate partners, respectively [5].

Women and girls are faced with physical, emotional, and sexual violence that threatens their safety and livelihood; disrupts their social structures and relationships [8, 9]. IPV is a serious, preventable public health problem that affects millions of women [10]. Evidence showed that sexually abused women often experience psychological, physical, economic, and social consequences such as depression, anxiety, sexual addiction, posttraumatic distress disorder, and substance abuse [11, 12]. Besides, IPV had imposed significant health impacts ranging from mild discomfort to extreme injury, abortion, anxiety, depression, post-traumatic illness, and death [11, 13].

Despite the international declaration of women's rights and national law to uphold the rights of women and girls enshrined in the constitution, IPV remains the commonest problem in Ethiopia [14–16]. A prior study conducted in Ethiopia found that 3 out of 4 women experience IPV in their lifetime [17]. Previous studies showed that household wealth status, women's age, residence, women's education, husband education, and parity were significant predictors of IPV [15, 18, 19]. Women from poor households rural resident women and uneducated women are more likely to experience intimate partner violence [20].

The distribution of education, wealth index, fertility, and empowerment of women differed significantly across Ethiopia's regions [21]. Rural people account for an estimated 80% of the population [22]. s with health indicators, education differed significantly across Addis Ababa, Dire-Dawa, and Harari regions with the highest rate of literacy, whereas Afar, Benishangul-Gumuz, and Somali have the lowest level of literacy. Also, women have limited access to health care facilities and health knowledge in rural and less developed regions (Afar, Somali, Benishangul-Gumuz, and Gambella) compared to more developed regional states (Amhara, Oromia, and Tigray) [23]. The more intensive labor such as plowing, trading, constructing, and harvesting is the duty of men in the countryside [24]. Women are more accountable for the household's domestic labor, such as cooking, gathering goods, and household care. Compared to girls, education is still more stressed for boys and also provided more leeway to social activities over girls, while enrollment rates for girls in education are growing [25].

The prevalence of intimate partner violence has varied within and across the country [26]. The presence of IPV indicates poor women’s empowerment in the community [27]. There are several studies conducted in Ethiopia about the prevalence of IPV and associated factors [28–30]. However, the results of these studies are unable to capture the spatial distribution and determinants of intimate partner violence across the country. Therefore, the current study aimed to investigate the spatial distribution and determinants of intimate partner violence among women of reproductive age in Ethiopia. The results of this study could help to identify significant hotspot areas of intimate partner violence and design evidence-based public health interventions targeting the susceptible groups.

## Methods

### Data source

This study was based on the 2016 Ethiopian Demographic and Health Survey (EDHS) data. EDHS is a nationally representative survey conducted in every five years interval in Ethiopia. Ethiopia has nine regional states (Afar, Amhara, Benishangul-Gumuz, Gambela, Harari, Oromia, Somali, Southern Nations, Nationalities, and People’s Region (SNNP) and Tigray) and two Administrative Cities (Addis Ababa and Dire-Dawa). A stratified two-stage cluster sampling technique was employed to select the study participants. At the first stage, a total of 645 Enumeration Areas (EAs) were selected. In the second stage, on average 28 households per EA were selected. Overall, for EDHS 2016 a total of 18,008 households were chosen and 16,583 eligible women in the selected household were identified. For this study, a total weighted sample of 6090 women were included. The detailed sampling procedure has been presented in the full report EDHS 2016 [31].

### Measurements of variables

#### Outcome variable

Having experienced IPV was the outcome variable for this study. Women were asked whether or not experienced any of the specified acts of physical, sexual, or emotional violence committed by their current husband/partner or most recent husband/partner in the 12 months preceding the survey was considered as experienced IPV, and if not were considered as never experienced IPV [10].

#### Independent variables

The data sources we used for this study were EDHS data and this data has hierarchical nature. The independent variables were collected at two levels (at individual and community levels). At the individual level, variables such as women's education, religion, sex of household head, women age, women occupation, wealth status, family size, number of unions, husband education, and media exposure were included. At level two, variables such as residence, region, community media exposure, community women employment, community women education, and community poverty were considered. The community-level variables considered in this study were from two sources. First, variables as collected without manipulation such as residence and region. In EDHS except for region and place of residence, there is no variable collected at the community level. Therefore, we generate community media exposure, community women employment, community women education, and community poverty by aggregating women's education, women occupation, media exposure, and wealth index at cluster/EA levels. Then these variables were categorized as high or low based on the national media values since these were not normally distributed.

## Data management and analysis

### Spatial analysis

ArcGIS version 10.6 and SaTScan version 9.6 statistical software were used to explore the spatial distribution and to identify the hotspot areas of intimate partner violence. The spatial global autocorrelation (Global Moran's I) was used to determine whether intimate partner violence was randomly distributed or not [29]. Moran's I is a spatial statistic used to measure autocorrelation in space by taking the entire data set and generating a single output value ranging from -1 to + 1. A statistically significant Moran's I value (p < 0.05) indicates that the spatial distribution of intimate partner violence is non-random and suggests the existence of spatial autocorrelation. Besides, Getis-OrdGi * statistical hotspot analysis was done to identify significant hotspot and cold spot areas of intimate partner violence [32]. The Bernoulli based model spatial scan statistical analysis was conducted to identify significant primary and secondary clusters of intimate partner violence. The SaTScan uses a circular scanning window that goes across the region of the study. Women who had experienced intimate partner violence were considered cases while those who had not experienced intimate partner violence were taken as controls to fit the Bernoulli model. The default overall spatial cluster size of < 50% of the population was used as an upper limit, allowing for the identification of small and large clusters and excluding clusters that contained more than the maximum limit. A likelihood ratio test statistic and the p-value were used to determine significant clusters for each possible cluster. The most likely performing cluster was the scanning window with a maximum likelihood. The primary and secondary clusters were established and ranked based on their likelihood test, based on 999 replicates from Monte Carlo [33].

The Kriging spatial interpolation technique was applied to predict the prevalence of IPV in un-sampled/unmeasured areas based on the values observed from sampled areas. There are various deterministic and geostatistical interpolation methods [34]. For this study, the Ordinary Kriging spatial interpolation method was used since it had a smaller residual and root mean square error.

### Multilevel analysis

The data were weighted using sampling weight, primary sampling unit, and strata before any statistical analysis to restore the representativeness of the survey and to take into account the sampling design to get reliable statistical estimates. Descriptive and summary statistics were conducted using STATA version 14 software. The EDHS data has hierarchical nature and women are nested within a cluster and we expect that women within the same cluster may be more similar to each other than women in the rest of the country. This violates the assumption of the traditional regression model which is the independence of observations and equal variance across clusters. This implies that the need to take into account the between cluster variability by using an advanced model. Therefore, a multilevel random intercept logistic regression model was fitted to estimate the association between the individual and community level variables and the likelihood of experiencing intimate partner violence. Model comparison was done based on Deviance (The negative 2 log-likelihood (− 2LL)) since the models were nested. Likelihood ratio test, Intra-class Correlation Coefficient (ICC), Median Odds Ratio (MOR), and Proportional Change in Variance (PCV) were computed to measure the variation between clusters. ICC quantifies the degree of heterogeneity of intimate partner violence between clusters (the proportion of the total observed individual variation in intimate partner violence that is attributable to between cluster variations).

ICC = ϭ^2^/ (ϭ^2^ + π^2^/3) [35], but MOR is quantifying the variation or heterogeneity in outcomes between clusters and is defined as the median value of the odds ratio between the cluster at high risk of experiencing intimate partner violence and cluster at lower risk when randomly picking out two clusters (EAs) [36].

$${\text{MOR}} = {\text{ exp }}(\sqrt {2*\partial 2*0.6745} ) \sim {\text{MOR}} = {\text{exp }}(0.95*)$$

$\partial$ ^2^ indicates that cluster variance.

PCV measures the total variation attributed to individual-level factors and community-level factors in the multilevel model as compared to the null model.

$$PCV = \frac{{var \left( {null model} \right) - var \left( {full model} \right)}}{{var \left( {null model} \right)}}$$

Multilevel random intercept logistic regression was used to analyze factors associated with intimate partner violence at two levels to take into account the hierarchical structure of the data, at individual and community (cluster) levels. Four models were constructed for the multilevel logistic regression analysis. The first model (a multilevel random intercept logistic regression model without covariates) was an empty model without any explanatory variables, to determine the extent of cluster variation on intimate partner violence. The second model (determined the association between the individual level predictors and intimate partner violence) was adjusted with individual-level variables; the third model (determined the association between community-level variables and intimate partner violence) was adjusted for community-level variables while the fourth (individual and community level model) was fitted with both individual and community level variables simultaneously. The final model (a model with individual and community level factors) was chosen since it had the lowest deviance.

Variables with p-value ≤ 0.2 in the bi-variable analysis for both individual and community-level factors were fitted in the multivariable model. Adjusted Odds Ratio (AOR) with a 95% Confidence Interval (CI) and p-value < 0.05 in the multivariable model were used to declare significant predictors of intimate partner violence. Multi-collinearity was also checked using the variance inflation factor (VIF) which indicates that there is no multi-collinearity since all variables have VIF < 5 and tolerance greater than 0.1.

### Ethical consideration

Permission for data access was obtained from major demographic and health survey through an online request from http://www.dhsprogram.com. The data used for this study were publicly available with no personal identifier. We received the authorization letter from The Demographic and Health Surveys (DHS) Program. The IRB-approved procedures for DHS public-use datasets do not in any way allow respondents, households, or sample communities to be identified. There are no names of individuals or household addresses in the data files. The geographic identifiers only go down to the regional level (where regions are typically very large geographical areas encompassing several states/provinces). Each enumeration area (Primary Sampling Unit) has a PSU number in the data file, but the PSU numbers do not have any labels to indicate their names or locations. In surveys that collect GIS coordinates in the field, the coordinates are only for the enumeration area (EA) as a whole, and not for individual households, and the measured coordinates are randomly displaced within a large geographic area so that specific enumeration areas cannot be identified.

## Results

### The characteristics of respondents

A total of 6090 women were included in the study. Of these, 3207 (52.7%) of women did not have formal education and 925 (15.6%) of the women attained secondary education or higher. About 1106 (18.2) of the women were from the poorest household and 2502 (41.1%) had media exposure. Nearly three-fourth (76.1%) of household heads were males and about 3580 (58.8%) of the women were participated in making decisions (Table 1). About 2281 (37.5%) and 1477 (24.3%) are living in the Oromia and Amhara regions, respectively. Regarding community media exposure and community poverty, about 47.3% of the women were from community with high media exposure and 51.9% from high community poverty (Table 2).

**Table 1:** Individual level characteristics of reproductive age women in Ethiopia, 2016

Characteristics	Category	Weighted frequency (N = 6090)	Percentage
Age of respondent	15–19	982	16.1
	20–24	1027	16.9
	25–29	1277	21.0
	30–34	1098	18.0
	35–39	814	13.4
	40–44	498	8.2
	45–49	392	6.5
Religion	Orthodox	2652	43.6
	Catholic	39	0.6
	Protestant	1400	23.0
	Muslim	1905	31.3
	Traditional	51	0.9
	Others	41	0.7
Women education	No	3207	52.7
	Primary	1958	32.1
	Secondary or higher	925	15.6
Sex of household head	Male	4635	76.1
	Female	1455	23.9
Wealth index	Poorest	1106	18.2
	Poorer	1170	19.2
	Middle	1195	19.6
	Richer	1167	19.2
	Richest	1451	23.8
Husband education	No education	2063	33.8
	Primary	1627	26.7
	Secondary or higher	2400	39.4
Number of unions	Once	4060	66.7
	More than once	2030	33.3
Media exposure	No	3588	58.9
	Yes	2502	41.1
Respondent working	No	3987	65.5
	Yes	2103	34.5
Women autonomous in making decisions	No	2510	41.2
	Yes	3580	58.8
Household size	1–4	2522	41.4
	5–7	2749	45.2
	8–10	725	11.9
	> 10	93	1.5

**Table 2:** Community level characteristics of reproductive age women in Ethiopia, 2016

Variable	Category	Weighted frequency	Percentage
Region	Tigray	414	6.8
	Afar	49	0.8
	Amhara	1477	24.3
	Oromia	2281	37.5
	Somali	183	3.0
	Benishangul	62	1.0
	SNNPRs	1275	20.9
	Gambela	16	0.3
	Harari	15	0.2
	Addis ababa	285	4.7
	Dire Dawa	33	0.6
Residence	Rural	4877	80.0
	Urban	1213	20.0
Community women education	Low	3380	55.5
	High	2710	44.5
Community poverty	Low	2932	48.1
	High	3158	51.9
Community women empowerment	Low	3071	50.5
	High	3011	49.5
Community media exposure	Low	3208	52.7
	High	2882	47.3

### Spatial analysis

**Fig. 1:**
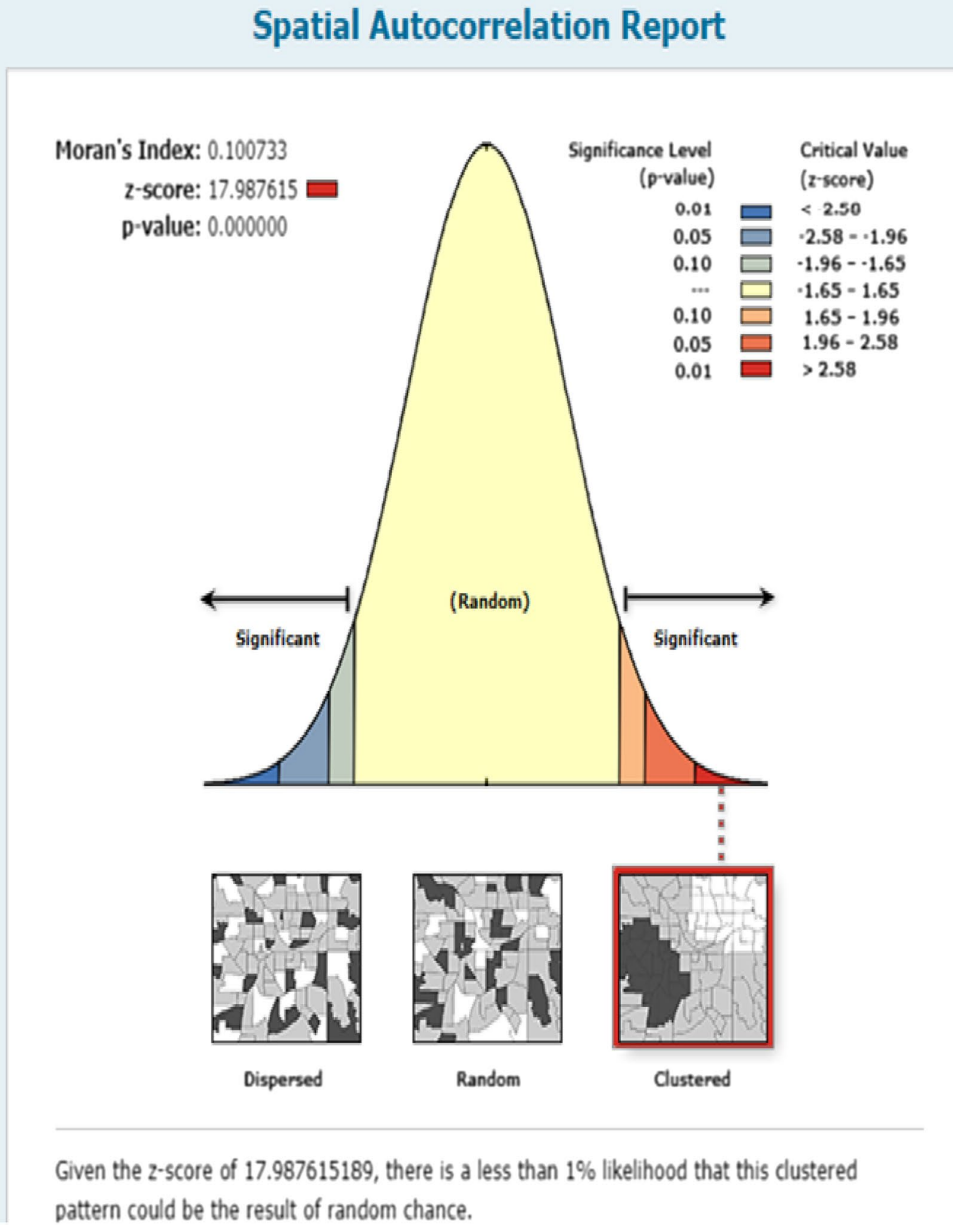
Global spatial autocorrelation of intimate partner violence among reproductive age women in Ethiopia, 2016

**Fig. 2:**
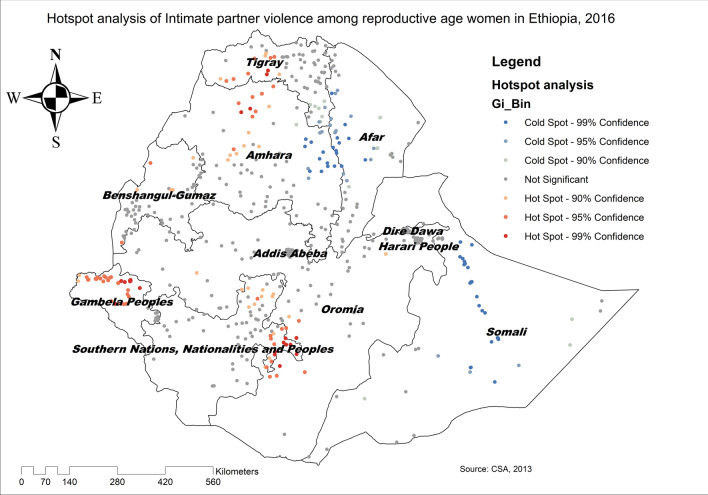
Hotspot analysis of intimate partner violence in Ethiopia, 2016 ( *Source*: CSA, 2013)

The overall national prevalence of IPV among reproductive-age women in Ethiopia was 33.5% (95% CI 32.1, 34.7). The spatial distribution of IPV was non-random in Ethiopia (Global Moran's I = 0.1, p-value < 0.0001) (Fig. 1). In the Getis Ord GI statistical analyses, the significant hotspot areas of IPV were located in the east SNNPRs, west Oromia, Gambella, north Amhara, and northwest Tigray regions, whereas significant cold spot areas of IPV were found in east Amhara, west Afar, and Somali regions (Fig. 2).

**Fig. 3:**
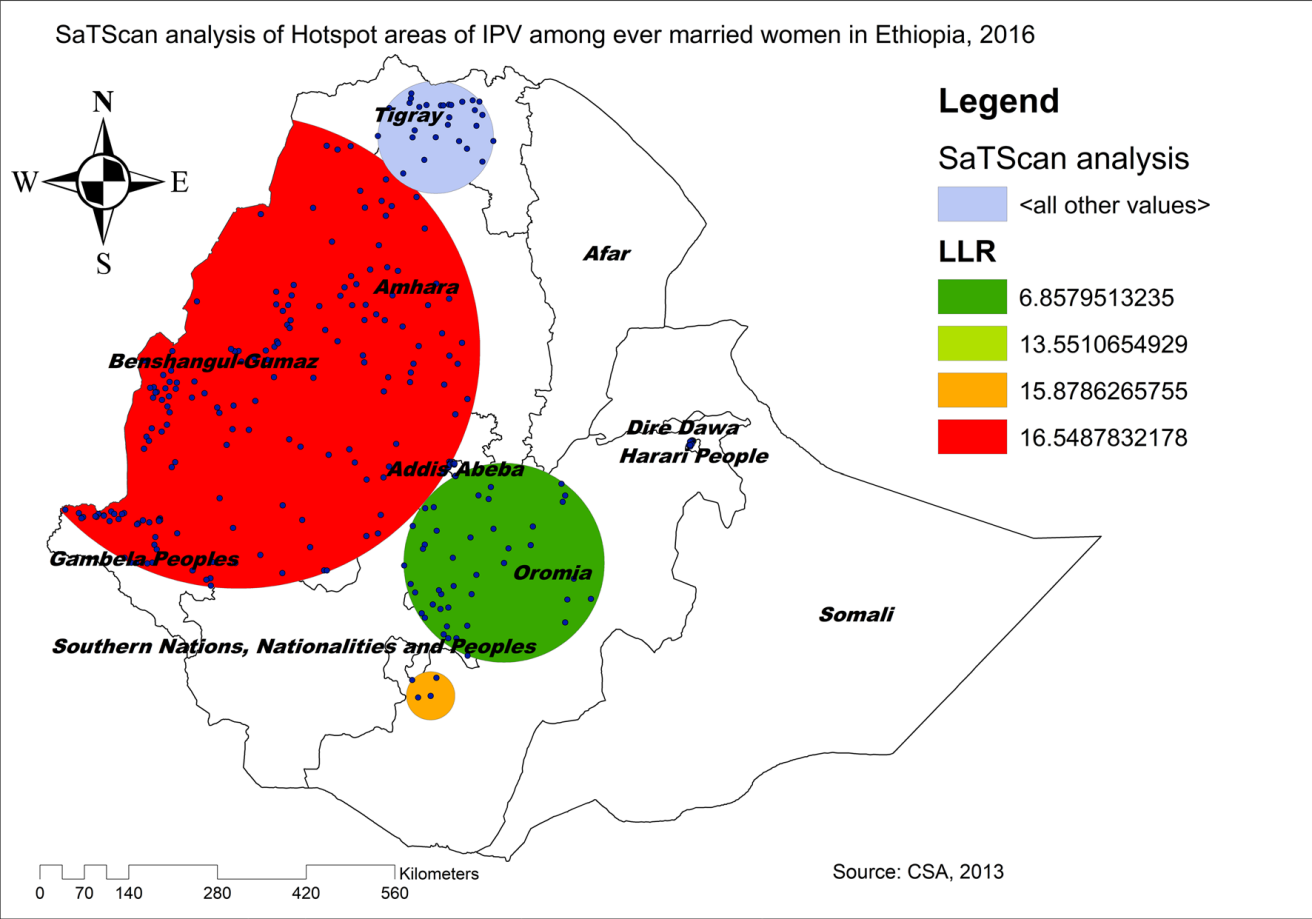
SaTScan analysis of hotspot areas of intimate partner violence in Ethiopia, 2016 ( *Source*: CSA, 2013)

**Fig. 4:**
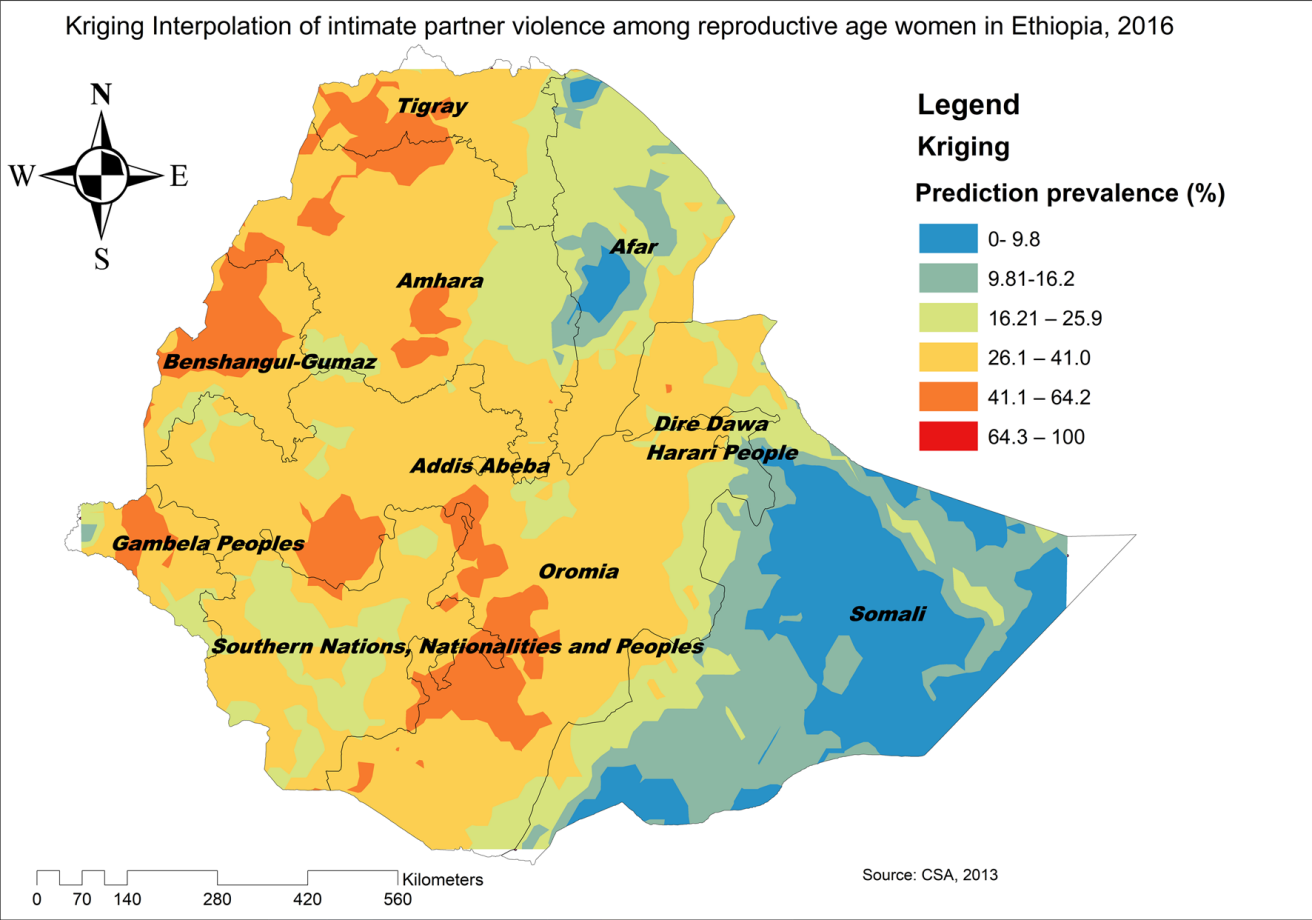
Kriging interpolation of intimate partner violence in Ethiopia, 2016 ( *Source*: CSA, 2013)

The SaTScan analysis identified a total of 192 significant clusters, of these 181 clusters were primary clusters located in Benishangul-Gumuz, Gambella, northwest Amhara and west Oromia regions centered at 10.637520 N, 35.719206 E with a radius of 373.97 km, a Relative Risk (RR) of 1.35 and a Log-Likelihood Ratio (LLR) of 16.55, at p < 0.001 (Table 3). This showed that women within the spatial window had 1.35 times higher risk of experiencing IPV than women outside the spatial window. The secondary clusters scanning window was located between the border area of the southwest Oromia, and north Tigray regions (Fig. 3). The Kriging interpolation identified northwest Tigray, northern and eastern Amhara, west Benishangul, east SNNPRs, and southwest Oromia regions as predicted high-risk areas of IPV while the Somali region was identified as predicted low prevalence of IPV (Fig. 4).

### Determinants of intimate partner violence

#### Random effect results

The ICC-value in the null model was 23% indicated that 23% of the total variability for IPV was attributable to the between group variation while the remaining 77% was explained by the between individual variation. Besides, the MOR was 2.56 indicated that, if we randomly select two women from two different clusters, women at the cluster with a higher risk of IPV had 2.56 times higher likelihood of experiencing IPV compared with women at cluster with a lower risk of IPV (Table 4). Moreover, Therefore, multilevel binary logistic regression analysis was mandatory to take in to account the clustering effect. A total of four models (null model, model with individual level variables, model with community level variables, and the final model that was model with both individual and community level variables) were fitted and the final model was the best-fitted model for the data since it had the lowest deviance value.

**Table 3:** SaTScan analysis result of hotspot areas of intimate partner violence in Ethiopia, 2016

Clusters	Enumeration areas identified	Coordinate/radius	population	case	RR	LLR	*p *Value
1 (181)	137, 364, 35, 244, 183, 36, 150, 559, 533, 246, 184, 320, 569, 65, 615, 88, 498, 548, 386, 515, 124, 256, 621, 457, 335, 541, 209, 324,415, 563, 407, 602, 409, 494, 349, 70, 595, 433, 259, 109, 416, 6, 203, 361, 581, 17, 317, 285, 508, 304, 165, 3, 161, 374, 462, 294,516, 395, 382, 431, 375, 474, 275, 193, 429, 175, 167, 169, 73, 248, 643, 280, 52, 403, 292, 24, 399, 558, 218, 531, 158, 234, 411, 279,456, 120, 512, 229, 350, 482, 163, 132, 555, 206, 62, 118, 517, 63, 47, 469, 549, 291, 114, 221, 231, 485, 640, 23, 38, 448, 106, 105,315, 327, 343, 346, 638, 567, 176, 265, 69, 426, 586, 510, 627, 312, 603, 10, 432, 152, 593, 104, 260, 177, 233, 119, 545, 592, 219, 262, 267, 507, 370, 199, 460, 423, 504, 296, 142, 326, 322, 536, 309, 489,446, 435, 174, 554, 572, 270, 46, 612, 628, 266, 284, 486, 13, 274, 299, 447, 147, 339, 417, 145, 463, 107, 608, 487, 31, 91, 100	(10.637520 N, 35.719206 E)/373.97 km	1385	508	1.31	16.55	0.00008
2 (4)	398, 21, 316, 182	(5.748741 N, 38.443060 E)/38.14 km	39	29	2.45	15.88	0.00014
3 (7)	357, 419, 288, 381, 495, 329, 321	(9.358685 N, 42.167437 E)/5.41 km	56	36	2.12	13.55	0.0011
4 (44)	562, 213, 619, 123, 524, 438, 26, 319, 522, 589, 149, 391, 578, 365, 452, 290, 472, 518, 54, 125, 12, 633, 14, 308, 529, 289, 576, 405, 217, 245, 609, 313, 286, 468, 420, 139, 216, 148, 122, 204, 297, 353, 83, 34	(7.634301 N, 39.484475 E)/156.86 km	345	137	1.33	6.86	0.353

**Table 4:** Random effect results and model comparison

Random effect	Model I	Model II	Model III	Model IV
Community variance (SE)	0.98(0.09)	0.72(0.11)	0.36(0.07)	0.48(0.09)
ICC (%)	23.0	17.95	9.8	12.6
PCV (%)	Reference	24.5	63.3	51.0
MOR	2.56	2.24	1.77	1.93
*Model fitness*				
Log likelihood	− 2824.12	− 2357.229	− 2752.752	− 2300.419
Deviance	5648.24	4714.46	5505.50	4600.83

**Table 5:** Multilevel logistic regression analysis of individual and community level factors associated with intimate partner violence in Ethiopia, 2016

Characteristics	Model 1	Model II (AOR with 95%)	Model III(AOR with 95%)	Model IV (AOR with 95%)
*Age category*				
15–19(ref)		1		1
20–24		1.82 (1.06, 3.13) *		1.87 (1.09, 3.20) **
25–29		1.90 (1.12, 3.20) *		2.00 (1.17, 3.41) *
30–34		1.94 (1.08, 3.48) *		2.02 (1.11, 3.69) *
35–39		2.13 (1.11, 4.10) *		2.21 (1.14, 4.31) *
40–44		2.01 (1.07, 3.76) *		2.10 (1.12, 3.94) *
45–49		2.62 (1.46, 4.70) *		2.79 (1.52, 5.10) *
*Place of residence*				
Urban		1		1
Rural		1.16 (0.72, 1.85)		1.04 (0.62, 1.75)
*Education*				
No education		1		1
Primary		1.05 (0.72, 1.89)		1.02 (0.77, 1.36)
Secondary or higher		0.52 (0.31, 0.87) *		0.57 (0.35, 0.91) **
*Wealth status*				
Poorest				1
Poor		0.93 (0.72, 1.20)		0.80 (0.61, 1.04)
Middle		0.97 (0.67, 1.40)		0.84 (0.57, 1.23)
Richer		0.80 (0.58, 1.12)		0.73 (0.51, 1.06)
Richest		0.63 (0.40, 0.98) *		0.58 (0.35, 0.97) *
*Marital status*				
Not in union		1		1
In union		1.39 (0.68, 2.84)		1.35 (0.66, 2.75)
*Women empowerment*				
Low		1		1
High		0.78 (0.62, 0.89) *		0.81 (0.63, 1.04)
*Number of household*				
1–4		1		1
5–7		0.94 (0.72, 1.22		0.94 (0.72, 1.23)
8–10		1.10 (0.69, 1.75)		1.13 (0.71, 1.79)
> 10		3.83 (1.47, 9.96) *		3.85 (1.41, 10.54) *
*Number of union*				
Once			1	1
More than once			1.13 (0.76, 1.67)	1.19 (0.87, 1.63)
*Region*				
Tigray			0.82 (1.08, 1.82)	0.86 (0.76, 1.82)
Afar			0.50 (0.28, 0.89) *	0.35 (0.18, 0.69) *
Amhara			1.02 (0.73, 2.06)	1.02 (0.56, 1.87)
Oromia			1.03 (0.8, 2.24)	1.03 (0.57, 1.83)
Somali			0.18 (0.09, 0.34) *	0.12 (0.06, 0.25) *
Benishangul			1.02 (0.59, 1.73)	0.87 (0.47, 1.67)
SNNPR			0.84 (0.57, 1.40)	0.71 (0.39, 1.26)
Gambela			1.17 (0.67, 2.01)	1.047 (0.53, 2.04)
Harari			1.71 (0.99, 2.93)	1.65 (0.86, 3.15)
Addis Ababa			1	1
Dire Dawa			1.04 (0.65, 1.67)	1.03 (0.58, 1.83)
*Community media exposure*				
Not exposed			1	1
Exposed			0.76 (0.59, 0.98) *	0.88 (0.85, 1.29)
*Community women empowerment*				
Low				
High			0.67 (0.52, 0.86) *	0.66 (0.49, 0.89) *
*Community poverty level*				
Low			1	1
High			0.89 (0.67, 1.19)	0.92 (0.65, 1.31)
*Educational level*				
Low educational attainment			1	1
High education attainment			0.90 (0.68, 1.20)	0.85 (0.62, 1.19)

AOR: adjusted odds ratio, CI: confidence interval

*p-value < 0.05, **p-value < 0.01

#### Fixed effect results

In the multivariable multilevel logistic regression analysis; women's age, women education, wealth index, region, family size, and community women empowerment were the significant determinants of IPV. Among the individual-level variables; mothers aged 20–24 and 25–29, 35–39, 40–45 and 45–49 years were 1.87 times (AOR = 1.87; 95% CI 1.09–3.20), 2.00 times (AOR = 2.00; 95% CI 1.17–3.41), 2.02 times (AOR = 2.02; 95% CI 1.11–3.69), 2.21 times (AOR = 2.21; 95% CI 1.14–4.31), 2.10 times (AOR = 2.10; 95% CI 1.12–3.94) and 2.79 times (AOR = 2.79; 95% CI 1.52–5.10) higher likelihood of experiencing IPV than women aged 15–19 years, respectively. The likelihood of experiencing IPV among women who attained secondary education or higher were decreased by 43% (AOR = 0.57; 95% CI 0.35–0.91) compared to women who did not have formal education. Women from the richest household had 42% (AOR = 0.58; 95% CI 0.35–0.97) decreased likelihood of experiencing IPV compared to those mothers from the poorest households. Women in the nuclear family (family size > 10) had 3.85 times (AOR = 3.85; 95% CI 1.41–10) higher likelihood of experiencing IPV compared to those women in family size of four and less.

Among community level variables, the likelihood of experiencing IPV among women living in Afar and Benishangul regions were decreased by 65% (AOR = 0.35; 95% CI 0.183–0.69) and 88% (AOR 0.12; 95% CI 0.06–0.25) compared to women in Addis Ababa, respectively. The likelihood of IPV among women in the community with higher women empowerment were decreased by 34% (AOR = 0.66; 95% CI 0.49–0.89) than women in the community with lower community empowerment (Table 5).

## Discussion

The spatial distribution of IPV was significantly varied across the country. The significant hotspot areas of IPV were located in the Benishangul-Gumuz, Gambella, northwest Amhara, and west Oromia regions. This could be due to the difference in cultural belief and misconceptions about IPV as husband’s have the right for beating, choking and forced sex of their wife [37]. Besides, the geographic variation in IPV might be attributable to the difference in awareness and attitude of husbands/partners toward negative consequent of women violence [38]. Moreover, IPV is closely linked with poor women's education, and women empowerment, the regional variation in education and women autonomy in the border areas might be the reason for the spatial variation [39].

In the multilevel analysis; women age, women education, wealth status, family size, region, and community women empowerment were significant determinants of IPV. The likelihood of experiencing IPV among women from the richest household were lower compared to women from a poor household. This is consistent with studies reported in Uganda [11], Nepal [13] and Philippines [40], This could be explained by the fact that women in poor households are more likely to be vulnerable to intimate partner violence because of their economic dependence on meeting their basic needs and are exposed to abuse, while rich women are more autonomous in decision-making [17, 41].

Age of women was found significant predictors of intimate partner violence. Advanced age was significantly associated with higher likelihood of experiencing intimate partner violence than women aged less than 20 years. This was supported by a previous study [42]. This could be due to the fact that advanced age women have large family size and this could increase the burden of women such as workload, and economic burden to meet the basic needs of their children, that can increase the risk of marital dispute [15]. Besides, advanced age are associated with increased household hardship, increased arguments over the partner’s inability to provide for the family, and decreased likelihood of relationship dissolution as they have too many children [43].

Women had secondary education or higher were less likely to experience of intimate partner violence than women who had no formal education. It is consistent with studies reported in Bangladesh [44], and Vietnam [45]. It might be due to the fact that women with secondary education or higher have improved access to information towards women empowerment or they may have less acceptances for partner violence than uneducated women [46, 47]. Moreover, women with higher level of education are less tolerant to beating, choking, and forced sex and their acceptance and tolerance towards husband’s mistreatment and control over the wife markedly declined as the education level of the women improved [48, 49].

The likelihood of experiencing intimate partner violence increases as family size increases. As majority of the people in Ethiopia are living under the poverty line with high rate of unemployment, which creates pressure on men to discharge their responsibilities as head of the household and could create poor interaction with their wife [50]. Besides, it could be due to the fact that families with lower number of household member may find it easier to meet their basic needs than families with a larger household member [51]. Therefore, the resources are lacking and when facing numerous family needs that are echoed by the wife, the husband may resort to violence [52].

Women from the community with a higher women empowerment community has been significantly lower risk of experiencing intimate sexual violence than a low women empowerment community. This is consistent with study findings in Bangladesh [53] and Peru [54]. This might be due to the reason that women who are empowered are able to fight for their rights and will not accept men to fully dictate to them which could result in sexual, physical or emotional violence [55]. Besides, in Ethiopia, majority of the cultures considered women to be subordinated or controlled by men and therefore, women in community with high women empowerment are not depend men for their lives and tend to resist some of the decisions of men which may bring about intimate partner violence [56].

The study has several strengths. First, the study was based on the nationally representative national EDHS survey which were weighted and it can be generalizable to the reproductive-age women in Ethiopia. Second, the use of GIS and SaTScan statistical analyses helped to detect specific and statistically important IPV hotspot areas to design effective public health interventions. The findings of this study should be interpreted considering the following limitations. First, the SaTScan detects only circular clusters but it can not detect the irregular clusters. Second, the kriging interpolation technique assumes that the space being studied is stationary and the joint probability does not change throughout the study area, due to these the interpolated values might be higher or lower than the real values in non-stationary areas. Besides, the EDHS survey did not include variables at the community level, such as community norms, culture, and beliefs that are closely linked with IPV. Moreover, the data were obtained based on the report of mothers or caregivers and may have the potential of social desirability and recall bias, because IPV is not socially acceptable, while CSA argues that substantial attempts have been made to reduce this, primarily by thorough training of data collectors, hiring skilled data collectors and managers, which may misrepresent our results.

## Conclusion

The spatial distribution of intimate partner violence was significantly varied across the country with the significant hotspot areas located in the Benishangul-Gumuz, Gambella, North West Amhara, and west Oromia regions. Advanced maternal age and large family size were significantly associated with an increased likelihood of experiencing intimate partner violence whereas women who had secondary education, richest wealth status, and community with high women empowerment were significant predictors of decreased risk of experiencing intimate partner violence. This finding highlights the need for designing spatially targeted public health programs and interventions to the identified significant hotspot areas of IPV to reduce the incidence of IPV in these areas. Public health interventions like enhancing women's empowerment in the community to decide on their health, promoting women's education and financial resources since it has the potential to enhance the decision-making capabilities of women to reduce intimate partner violence. However, much to be done on promoting women education in Ethiopia it is needed to scale up the programs to prevent intimate partner violence.

## Data Availability

The data used for this study are publicly available and can access it from https://www.dhsprogram.com/data/dataset_admin/login_main.cfm.

## Abbreviations

AOR: Adjusted odds ratio
CI: Confidence interval
CSA: Central statistical agency
DHS: Demographic health survey
EA: Enumeration area
EDHS: Ethiopian demographic health survey
GIS: Geographic information system
ICC: Intra-cluster correlation coefficient
LLR: Log-likelihood ratio
LR: Likelihood ratio
MOR: Median odds ratio
PCV: Proportional change in variance
PHC: Population and housing census
SNNPRs: Southern nations and nationality people regional state

## References

[1] Dahlberg LL, Krug EG (2006). Violence a global public health problem. Ciência & Saúde Coletiva.

[2] Leonardsson M, San SM (2017). Prevalence and predictors of help-seeking for women exposed to spousal violence in India–a cross-sectional study. BMC Women's Health.

[3] Mancini JA, Nelson JP, Bowen GL, Martin JA (2006). Preventing intimate partner violence: A community capacity approach. J Aggress Maltreat Trauma.

[4] World Health Organization (2012). Understanding and addressing violence against women: Intimate partner violence.

[5] Garcia-Moreno C, Jansen HA, Ellsberg M, Heise L, Watts CH (2006). Prevalence of intimate partner violence: findings from the WHO multi-country study on women's health and domestic violence. The Lancet.

[6] Ismayilova L (2015). Spousal violence in 5 transitional countries: a population-based multilevel analysis of individual and contextual factors. Am J Public Health.

[7] King JD, Endeshaw T, Escher E, Alemtaye G, Melaku S, Gelaye W (2013). Intestinal parasite prevalence in an area of Ethiopia after implementing the SAFE strategy, enhanced outreach services, and health extension program. PLoS Negl Trop Dis.

[8] Leatherman J. Sexual violence and armed conflict: Polity; 2011.

[9] Black M, Basile K, Breiding M, Smith S, Walters M, Merrick M, et al. National intimate partner and sexual violence survey: 2010 summary report. 2011.

[10] Basile KC, Black MC, Breiding MJ, Chen J, Merrick MT, Smith SG, et al. National intimate partner and sexual violence survey; 2010 summary report. 2011.

[11] Ogland EG, Xu X, Bartkowski JP, Ogland CP (2014). Intimate partner violence against married women in Uganda. J Family Violence.

[12] CSACEa. I. Ethiopia Demographic and Health Survey. Addis Ababa, Ethiopia, and Rockville , maryland, USA: CSA and ICF. 2016.: 2016.

[13] Atteraya MS, Gnawali S, Song IH (2015). Factors associated with intimate partner violence against married women in Nepal. J Interpers Violence.

[14] Azene ZN, Yeshita HY, Mekonnen FA (2019). Intimate partner violence and associated factors among pregnant women attending antenatal care service in Debre Markos town health facilities, Northwest Ethiopia. PLoS ONE.

[15] Abeya SG, Afework MF, Yalew AW (2011). Intimate partner violence against women in western Ethiopia: prevalence, patterns, and associated factors. BMC Public Health.

[16] Alebel A, Kibret GD, Wagnew F, Tesema C, Ferede A, Petrucka P (2018). Intimate partner violence and associated factors among pregnant women in Ethiopia: a systematic review and meta-analysis. Reprod Health.

[17] Montagu D, Yamey G, Visconti A, Harding A, Yoong J (2011). Where do poor women in developing countries give birth? A multi-country analysis of demographic and health survey data. PLoS ONE.

[18] Makayoto LA, Omolo J, Kamweya AM, Harder VS, Mutai J (2013). Prevalence and associated factors of intimate partner violence among pregnant women attending Kisumu District Hospital. Kenya Mat Child Health J.

[19] Walton-Moss BJ, Manganello J, Frye V, Campbell JC (2005). Risk factors for intimate partner violence and associated injury among urban women. J Commun Health.

[20] Azevêdo ACDC, Araújo TVBD, Valongueiro S, Ludermir AB (2013). Intimate partner violence and unintended pregnancy: prevalence and associated factors. Cadernos de saude publica..

[21] Ahmed S, Creanga AA, Gillespie DG, Tsui AO (2010). Economic status, education and empowerment: implications for maternal health service utilization in developing countries. PLoS ONE.

[22] Kloos H, Adugna A. The Ethiopian population: growth and distribution. Geogr J. 1989:33–51.

[23] Deressa T, Hassan RM, Ringler C. Measuring Ethiopian farmers' vulnerability to climate change across regional states: Intl Food Policy Res Inst; 2008.

[24] Gebre GG, Isoda H, Rahut DB, Amekawa Y, Nomura H, editors. Gender differences in the adoption of agricultural technology: the case of improved maize varieties in southern Ethiopia. Women's studies international forum; 2019: Elsevier. 10.1016/j.wsif.2019.102264 PMC6894305 31853161

[25] Tyre P. The trouble with boys: a surprising report card on our sons, their problems at school, and what parents and educators must do: Harmony; 2008.

[26] Fontes KB, Alarcão ACJ, Nihei OK, Pelloso SM, Andrade L, de Barros Carvalho MD (2018). Regional disparities in the intimate partner sexual violence rate against women in Paraná State, Brazil, 2009–2014: an ecological study. BMJ Open.

[27] Gracia E, López-Quílez A, Marco M, Lladosa S, Lila M (2015). The spatial epidemiology of intimate partner violence: do neighborhoods matter?. Am J Epidemiol.

[28] Deyessa N, Berhane Y, Alem A, Ellsberg M, Emmelin M, Hogberg U (2009). Intimate partner violence and depression among women in rural Ethiopia: a cross-sectional study. Clin Pract Epidemiol Mental Health.

[29] Deyessa N, Berhane Y, Ellsberg M, Emmelin M, Kullgren G, Högberg U (2010). Violence against women in relation to literacy and area of residence in Ethiopia. Glob Health Action.

[30] Abate BA, Wossen BA, Degfie TT (2016). Determinants of intimate partner violence during pregnancy among married women in Abay Chomen district, Western Ethiopia: a community based cross sectional study. BMC women's health.

[31] ICF CSACEa. Ethiopia Demographic and Health Survey 2016 Addis Ababa, Ethiopia, and Rockville , maryland, USA: CSA and ICF. 2016

[32] Tsai P-J, Lin M-L, Chu C-M, Perng C-H (2009). Spatial autocorrelation analysis of health care hotspots in Taiwan in 2006. BMC Public Health.

[33] Kulldorff M. SaTScanTM user guide. Boston; 2006.

[34] Bhunia GS, Shit PK, Maiti R (2018). Comparison of GIS-based interpolation methods for spatial distribution of soil organic carbon (SOC). J Saudi Soc Agric Sci.

[35] Rodriguez G, Elo I (2003). Intra-class correlation in random-effects models for binary data. Stata J.

[36] Merlo J, Chaix B, Ohlsson H, Beckman A, Johnell K, Hjerpe P (2006). A brief conceptual tutorial of multilevel analysis in social epidemiology: using measures of clustering in multilevel logistic regression to investigate contextual phenomena. J Epidemiol Commun Health.

[37] Kassa S (2015). Challenges and opportunities of women political participation in Ethiopia. J Glob Econ.

[38] Bazargan-Hejazi S, Medeiros S, Mohammadi R, Lin J, Dalal K (2013). Patterns of intimate partner violence: a study of female victims in Malawi. J Injury Violence Res.

[39] Organization WH. Increasing access to health workers in remote and rural areas through improved retention: global policy recommendations: World Health Organization; 2010. 23741785

[40] Hindin MJ, Adair LS (2002). Who's at risk? Factors associated with intimate partner violence in the Philippines. Soc Sci Med.

[41] Acharya DR, Bell JS, Simkhada P, Van Teijlingen ER, Regmi PR (2010). Women's autonomy in household decision-making: a demographic study in Nepal. Reprod Health.

[42] Azene ZN, Yeshita HY, Mekonnen FA. Intimate partner violence and associated factors among pregnant women attending antenatal care service in Debre Markos town health facilities, Northwest Ethiopia. PloS one. 2019;14(7). 10.1371/journal.pone.0218722 PMC6602189 31260469

[43] Miller E, Levenson R, Herrera L, Kurek L, Stofflet M, Marin L (2012). Exposure to partner, family, and community violence: Gang-affiliated Latina women and risk of unintended pregnancy. J Urban Health.

[44] Rahman M, Hoque MA, Makinoda S (2011). Intimate partner violence against women: Is women empowerment a reducing factor? A study from a national Bangladeshi sample. J Family Violence.

[45] Vung ND, Ostergren P-O, Krantz G (2008). Intimate partner violence against women in rural Vietnam-different socio-demographic factors are associated with different forms of violence: need for new intervention guidelines?. BMC Public Health.

[46] Madeira JL (2011). Woman scorned: resurrecting infertile women's decision-making autonomy. Md L Rev.

[47] Lamichhane P, Puri M, Tamang J, Dulal B (2011). Women's status and violence against young married women in rural Nepal. BMC Women's Health.

[48] Yount KM, Carrera JS (2006). Domestic violence against married women in Cambodia. Soc Forces.

[49] Horsman J. Too scared to learn: women, violence, and education: Routledge; 2013.

[50] Erulkar A. Early marriage, marital relations and intimate partner violence in Ethiopia. Int Perspect Sex Reprod Health. 2013:6–13. 10.1363/3900613 23584463

[51] Gennetian LA, Castells N, Morris PA (2010). Meeting the basic needs of children: does income matter?. Child Youth Serv Rev.

[52] Garikipati S (2008). The impact of lending to women on household vulnerability and women’s empowerment: evidence from India. World Dev.

[53] Dalal K, Dahlström Ö, Timpka T. Interactions between microfinance programmes and non-economic empowerment of women associated with intimate partner violence in Bangladesh: a cross-sectional study. BMJ Open. 2013;3(12). 10.1136/bmjopen-2013-002941 PMC3855592 24319278

[54] Svec J, Andic T (2018). Cooperative decision-making and intimate partner violence in Peru. Popul Dev Rev.

[55] Leite TH, Moraes CLd, Marques ES, Caetano R, Braga JU, Reichenheim ME. Women economic empowerment via cash transfer and microcredit programs is enough to decrease intimate partner violence? Evidence from a systematic review. Cadernos de saude publica. 2019;35:e00174818. 10.1590/0102-311X00174818 31508698

[56] Kwagala B, Wandera SO, Ndugga P, Kabagenyi A (2013). Empowerment, partner’s behaviours and intimate partner physical violence among married women in Uganda. BMC Public Health.

